# Supplementary material for: Are carbon emissions trading and green financial instruments synergistic? -Comprehensive quantitative research based on content analysis
Source: PLoS One. 2024 Mar 7;19(3):e0298601. doi: 10.1371/journal.pone.0298601 (PMC10919601; doi:10.1371/journal.pone.0298601)
Supplement: S1 Data — (ZIP) [file pone.0298601.s001.zip › Data/地级市Z_F工作报告.zip!/░▓╗╒/╬▀║■╩╨/└┤╫╘╣1⁄2╓┌║┼í╛┬φ┐╦╩2╛▌═°í┐_2016.docx]

政 府 工 作 报 告

——2016年3月1日在芜湖市第十五届人民代表大会第六次会议上

市长 潘朝晖

各位代表：

现在，我代表市人民政府，向大会报告工作，请予审议，并请市政协委员和其他列席人员提出意见。

一、2015年及“十二五”时期工作回顾

过去的一年，在中共芜湖市委的坚强领导下，全市人民积极应对复杂多变的宏观经济环境，主动适应经济发展新常态，全力打造经济和城市两个升级版，保持了经济社会健康较快发展的良好态势，较好完成了市十五届人大四次会议确定的任务，基本实现了“十二五”规划目标。

全市地区生产总值2457亿元，比上年增长10.3%；居民人均收入23998元，增长9.3%；财政收入470亿元，增长10.3%；城镇新增就业8万人，城镇登记失业率3.3%；单位生产总值能耗下降7.1%，可吸入颗粒物年平均浓度下降15微克/立方米，超额完成“十二五”节能减排任务。成功创建全国文明城市。

一年来，我们主要做了以下几个方面的工作。

一是坚持以项目引领产业提升。出台了促进经济持续健康发展22条意见，深化“一企一组一策”帮扶机制。兑现税收政策优惠70.4亿元，兑现企业扶持资金22亿元，发放稳岗和促进就业补贴2.1亿元，减缴缓缴社保费6.5亿元，减少涉企收费1.7亿元。飞尚非金属在港交所上市，新增30家“新三板”挂牌企业。全市直接融资283.4亿元。落实“政银担合作”贷款21亿元。金融机构净增贷款309亿元。

省外亿元以上项目到位资金1150亿元、外商直接投资23亿美元，分别增长16.5%和14.8%；总量和增幅分别位居全省第二、第一。在建亿元项目1085个，其中新开工亿元以上项目482个。新增规模工业企业245家、规模服务业企业148家。民间投资占比达79%，提高2个百分点。

汽车及零部件、材料、电子电器、电线电缆四大支柱产业共实现增加值974亿元，增长12.5%。奇瑞集团战略转型成效显现，年销售额增长7.8%。海螺集团实现利润107亿元。59家企业实施兼并重组。实施163万吨水泥产能减量置换。无为成为全国特种电缆产业知名品牌创建示范区。

战略性新兴产业实现增加值413亿元，增长16.5%。实施省加快调结构转方式促升级“4105”行动计划。机器人、新能源汽车、现代农业机械获批省战略性新兴产业集聚发展基地。构建机器人产业体系，埃夫特年产万台机器人基地、翡叶伺服电机等项目建成，欧凯罗博特机器人、国家机器人产品质量监督检验中心等项目开工。健全新型显示和光电信息产业链，东旭光电液晶玻璃基板、信义电子玻璃、兴飞手机、辉灿智能产业基地等项目投产，东旭光电装备制造、融捷光电显示终端、中兴通讯智能配套基地、信义光伏玻璃五期等项目开工。新能源汽车产业集聚发展，奇瑞、宝骐新能源汽车和物流车销售2.2万辆，天弋锂电池一期、达尼特锂电池隔膜等项目投产，天量锂离子电池箱、馨联动力系统等项目开工。现代农业机械产业发展迅猛。通用航空产业初具雏形，中电科钻石飞机生产线正在安装调试，万户科技无人植保机、东西方航空部件生产线项目建成，航瑞航空发动机项目开工。新材料产业加速布局，格丰纳米环保材料、格力精密铸件等项目开工。

服务业实现增加值797亿元，增长11%；服务业比重提高4个百分点。新增4家省级现代服务业集聚区。银行、证券、保险、小贷、担保、住房金融、汽车金融、金融资产管理等金融业态发展良好。航运服务业集聚区水上停泊区、宇培速通物流10万平方米立体仓建成运营。我市成为全国物流标准化试点城市。新增限额以上电商企业21家；限额以上电商企业网上零售额增长73%；无为县获批国家级电子商务进农村综合示范县。530家传统企业入驻“阿里巴巴·芜湖产业带”平台。快递业务量增长48%。设立皖南快递产业园，快件分拨量占皖南的90%以上。银泰城、星隆国际城、柏庄时代广场、绿地新都会开业。方特四期东方神画、大砻坊科技文化园、新华联鸠兹老街东区建成。美好乡村旅游已形成13条精品线路。接待游客3368.8万人次，增长19.8%。举办了第四届中国国际动漫创意产业交易会。

基本完成农村土地承包经营权确权登记。开展了无为农村产权交易体系建设试点。种植超级稻80万亩。粮食增产3.6%。农作物耕种收综合机械化水平达65%，提高3.5个百分点。新增家庭农场927家、农民专业合作社225家。

二是加强城市规划建设。出台了控制性详细规划通则。完成了供电、移动通信基站布局、城市抗震防灾等专项规划编制和弋江路沿线城市设计、长江南岸沿线天际线及地块开发强度、中心城区特色街区类型与布局等课题研究。启动了“多规合一”试点。

推进江北新区产城融合发展。江北集中区70公里路网及水电气、污水处理设施建成运行，公共服务设施进一步完善。大龙湾片区起步区路网主体基本形成。江北城市公交班线达9条。无为经济开发区城东园区起步区加快建设。

合福高铁、宁安高铁及新火车站、汽车客运南站、铜南宣高速公路建成运营。沪渝高速公路峨山路互通、弋牧公路二期、新芜大道开工。芜申运河弋江桥、荆山桥改建竣工通车，三山东汇2万吨级泊位、裕溪口万吨级泊位改扩建工程完工。民航机场选址获批。实施城市交通提升六年行动计划，芜石路立交、黄山东路立交建成通车，建成7处行人过街通道。新增公交车336辆。

出台了促进房地产市场健康发展的意见，商品房销售保持平稳。完成棚户区改造256万平方米。融通棚改资金50.4亿元，实现投资42.6亿元。启动建设城市地下管线信息系统。在全省率先建成“全光网城市”。完成了板城垾一期、大官沟水系、无为万亩圩口堤防达标四期工程和22个老旧小区综合整治。住宅小区物业服务覆盖面达86%。城市管理进一步加强。

三是深化改革开放。完成了市、县区政府机构改革。建立了三级政府权责清单及动态调整制度。推行“互联网+政府服务”，政府权力和公共服务事项全部网上运行。完成了全市不动产统一登记职责机构整合和市本级发证工作。完成了机关事业单位工资制度和市级公务用车制度改革，启动养老保险制度改革。启动以市场监管为重点的综合监管、分类执法改革试点。我市成为全国创建社会信用体系建设示范城市。建立了市县一体化公共资源交易体系。推行“三证合一”、“先照后证”改革，在全省先行试发了“一照一码”营业执照。新登记市场主体3.1万户，增长25.9%。

国有企业改革不断深化。发布了一批政府和社会资本合作项目，城南过江隧道项目签约。公开了各级政府、部门及“三公”经费预决算信息。清理盘活财政存量资金20.1亿元。争取地方政府置换债136.8亿元、新增债14.2亿元。“营业税改征增值税”改革平稳运行。出台了农村金融综合改革实施意见。南陵太平村镇银行开业。推进工业用地市场化配置改革试点，实行工业用地弹性出让。

出台了全面深化医改试点重点工作推进方案。推进公立医院综合改革，3家区级医院落实药品零差率销售；建立以成本费用控制和患者满意度为主的绩效考核体系。实施基层医疗卫生服务能力三年提升计划，评选培养百名基层优秀医师。实行按项目付费为主的居民医疗保险支付方式。推行商业保险公司承办大病医疗补充保险。

芜湖出口加工区优化升级为综合保税区获批，实现当年验收、当年封关运行，跨境电商平台及进口商品直销中心基本建成。港口货物吞吐量1.2亿吨，增长10.7%；集装箱量50.2万标箱，增长24.6%。出口退（免）税30亿元，增长10.9%。出台了外贸促进政策，新增进出口实绩企业78家。实现进出口总值68.2亿美元，其中出口55.4亿美元。新增境外投资企业19家。实际对外投资5.6亿美元，位居全省第一。建立中德中小企业技术文化交流中心。芜湖县建成全国首个省级出口木质包装质量安全示范区。芜湖港获批进口粮食指定口岸、筹建进口肉类指定口岸。

四是激励创新创业。出台了关于进一步提升人才特区建设水平的15条意见，设立了3亿元人才工作专项资金、5亿元众创创业股权投资基金、4亿元政府投资引导基金、20亿元文化旅游投资基金。举办了皖江青年科技创新创业大赛、大学生专利创新大赛和职工专利创新竞赛、首届“双创”项目路演资本对接会。发展银湖创客岛、皖江云等众创空间。出台了应届高校毕业生免费入住公租房政策。

新增高新技术企业65家、省级企业技术中心11家、省级工程（技术）研究中心13家。中联重机获批现代农业装备国家地方联合工程研究中心。新增国家级博士后科研工作站3家、院士工作站3家、产业技术研究院6家、省市共建重点（工程）实验室5家。新增发明专利授权量1927件，增长1.3倍。万人有效发明专利拥有量13.6件，稳居全省第一。启动全国质量强市示范城市创建。新认定中国驰名商标6件、省著名商标74件、安徽名牌19个，“欢乐芜湖”商标通过国家工商总局注册。我市成为全国创新驱动助力示范市、国家知识产权质押融资试点城市。

五是保护生态环境。完成重点减排项目39个，3座污水处理厂投入运营，6条水泥熟料生产线完成脱硝治理。淘汰落后水泥产能660万吨，整合关闭减少矿山14家。补充耕地2.3万亩。落实大气污染综合整治行动计划取得成效。淘汰黄标车4902辆。推广新能源汽车4993辆。繁昌获批建设国家级循环经济示范县。

华电二期1000兆瓦扩建工程能源消费等量置换方案获批。160兆瓦地面光伏电站和无为48兆瓦风电场建成并网，新增分布式光伏发电备案100兆瓦。

新增绿地429万平方米，新建绿道100公里，完成造林12.1万亩、森林长廊工程示范段110公里。投入2.2亿元，用于省级美丽乡村示范村建设。推进“三线三边”环境治理，完成18家矿山复绿。芜湖县国家级生态县创建工作通过环保部技术评估。

六是维护社会和谐稳定。实施了33项民生工程，完成投入91.6亿元。扶贫建档立卡和对象需求、目标时限、措施、责任“四个清单”全面实施，2.3万贫困人口实现脱贫。

扩大优质教育覆盖面，北师大附属学校开学，优质学校办分校10个，新建学前教育和基础教育集团10个、名园长工作室11个。完成教育信息化三年行动计划，中小学宽带网络接入率100%。启动省级卫生城市创建。皖南康复医院住院综合楼完工，皖南精神卫生中心、儿童中医特色治疗中心开工。出生人口政策符合率达95%。公共文化、体育场馆全部免费开放，滨江文化艺术中心投入运营。文艺、新闻出版、广播电视、档案、地方志、科普、气象等工作得到加强。承办了全国击剑冠军赛。

城镇职工五项保险参保246.6万人次，增长7.1%。城乡居民养老保险、城镇居民医疗保险和新型农村合作医疗保险参保分别达166万人、104万人和210万人。完成就业技能培训2.1万人，新增省级技能大师工作室1家。企业退休人员月均养老金由1717元提高到1816元。失业保险金标准由每月676元提高到1013元。市区最低工资标准上调到每月1350元。有3万人次享受大病医疗补充保险待遇5849万元。出台城乡困难群众临时救助办法。为13.5万低保对象发放低保金5.3亿元。支付3.3万人次困难群众医疗救助款8738万元。在全国率先开展进城务工农民缴存住房公积金及住房贷款试点。新增5.9万人缴存住房公积金。发放公积金贷款31亿元，支持1.2万户家庭购房。

出台了培育社会组织、发展养老服务业的意见和市区社区工作者（网格员）管理办法。我市成为全国社区治理和服务创新实验区。完成了“六五”普法任务。开展法律援助服务2.6万人次。支持工会、共青团、妇联等群团组织发挥作用。民族、宗教、外事、侨务、对台、妇女儿童、老龄、残疾人等工作进一步加强，建成城市少数民族流动人口服务管理体系。出台了推进军民融合深度发展实施意见，组织了三级国防动员指挥机构演练。推进食品药品安全专项整治。深刻汲取镜湖区“10.10”重大事故教训，开展严执法、排隐患、强管理“铸安”行动百日攻坚，提升城市公共安全和安全生产管理水平。严厉打击违法犯罪活动，建设“平安芜湖”，社会治安和信访形势总体稳定。

去年，政府系统开展了“三严三实”专题教育。加强法治政府建设，完善了行政机关规范性文件制定程序规定、重大行政决策程序规定，清理了市政府及部门规范性文件。坚持机关联系基层、干部联系群众，推行“亲切服务”。8家服务机构入驻市民服务中心。将8个部门服务热线整合纳入“12345政府服务直通车”。建立健全重要文件学习解读，政府重大决策出台前向市委汇报、向人大报告、与政协协商等制度。办理人大议案2件、代表建议172件和政协提案271件。2件议案由市长办公会、市政府专题会议研究办理，实现议案提案办理由答复满意向过程满意、结果满意转变。强化审计监督和廉政风险防控，出台了加强政府系统廉政建设若干具体问题的规定，打造“用人、用钱、用地、用权”制度升级版。开展廉政建设巡查，对违反中央八项规定和省、市改进作风有关规定精神的行为坚决查处，对腐败分子坚决严惩。

各位代表！

“十二五”时期，是我市发展很不平凡的五年。经济社会发展在应对一系列风险挑战中取得了重大成就。

这五年，综合实力跨上了新台阶。主要经济指标总量稳居全省第二位。地区生产总值由1341亿元增加到2457亿元，年均增长12.5%，居全省第一位。人均地区生产总值突破1万美元。累计固定资产投资超过1万亿元，是“十一五”累计投资的2.5倍。规模工业增加值1442亿元，是2010年的1.9倍；产值超亿元企业由316户增加到890户。财政收入翻了一番多，年均增长16.5%。

这五年，结构调整取得了新突破。服务业占比止跌回升，三次产业比重由6.8∶63.5∶29.7调整为4.9∶62.7∶32.4。首位产业汽车和装备制造业增加值占规模工业比重由30.8%提升到46.2%。四大支柱产业增加值占规模工业比重达67.6%，对规模工业增长贡献率保持在80%以上。战略性新兴产业产值占规模工业比重由17.2%提升到23.3%。高新技术产业增加值占规模工业的比重由38.5%提升到50.5%。省级以上研发机构由120家增加到283家，高新技术企业由266家增加到441家。粮食总产143万吨，比2010年增产17万吨。民营经济占比由60.5%提高到68.5%，增长贡献率达95%。

这五年，城乡面貌发生了新变化。区划调整顺利实施，江北新区启动建设。市区面积由827平方公里扩大到1491平方公里；建成区面积达165平方公里。累计完成棚户区改造5.3万户。城镇化率由54.6%上升到62%。我市迈入高铁时代。公路里程由4810公里增加到10363公里。新建扩建神山等9个公园，新增绿地2217万平方米，建设森林长廊275公里，完成造林55.6万亩，森林覆盖率由13.7%上升到18.8%，我市成为国家园林城市。市区污水处理率由60%提高到91%。

这五年，改革开放迈出了新步伐。农村综合改革和统筹城乡综合配套改革持续推进。医药卫生体制改革走在全国前列。扩大优质教育资源，教育信息化全国领先。创新财税管理，地方金融组织体系更加完善。完成统计数据采集方式改革。推进了政府职能转变和机构改革。在全省率先建立政府权责清单、企业投资负面清单，建立基于大数据的网上审批服务、事中事后监管体系。形成社会服务管理信息化“芜湖模式”。累计实际利用外商直接投资82.7亿美元，实际对外投资8.4亿美元。芜湖港跻身亿吨大港，我市成为长三角城市经济协调会会员城市。

这五年，民生保障达到了新水平。滚动实施184项民生工程，财政累计投入371亿元。学前教育三年毛入园率由74.6%提高到88.6%，完成554所义务教育学校标准化建设。高中阶段毛入学率由84.8%提高到95.5%。累计新增就业41.5万人。城乡居民人均收入年均分别增长11.8%和13.5%。基本建成覆盖全体居民的社会保障体系。完成1045户渔民上岸任务。累计建成保障房7.1万套，有6.4万户享受住房保障。每千人口拥有医疗卫生机构床位5张、执业医师2.18名。建成了市规划展示馆、市博物馆。

成就来之不易，经验弥足珍贵。我们深切体会到，推进芜湖发展，一是必须坚持产业发展这一立市之本，狠抓项目建设不松手，多渠道扩大有效投入，全力实施创新驱动发展战略，聚焦有限目标，抢占行业制高点，积极构建产业新体系。二是必须坚持城市品质提升这一核心要求，优化城乡环境，提升城市能级，有序推进以人为核心的新型城镇化，全力提升城市核心竞争力。三是必须坚持人才优先这一主战略，牢固树立人才是第一资源的观念，以引进培养高层次人才为重点，以优化人才发展环境为保障，构建全国一流的人才特区。四是必须坚持改革创新这一优良传统，崇尚创新、宽容失败、支持冒险、鼓励冒尖，释放改革活力，打造开放高地。五是必须坚持改善民生这一根本目的，从解决群众最关心最直接最现实的利益问题入手，履行政府职责，推进共享发展。六是必须坚持苦干实干这一过硬作风，各级领导干部以身作则，团结带领广大群众锐意进取、主动作为、科学作为，不断开创芜湖发展新局面。

各位代表！

“十二五”成果的取得，是中央、省委省政府和市委科学决策、坚强领导的结果，是市人大及其常委会和市政协强化监督、社会各界大力支持的结果，是全市人民万众一心、艰苦奋斗的结果。我代表市人民政府，向各级人大代表、政协委员，向来芜湖创新创业的投资者和各类人才，向广大工人、农民、知识分子、干部、离退休老同志，向驻芜解放军指战员、武警官兵和政法干警，向各民主党派、各人民团体和各界人士，向关心支持芜湖发展的上级部门、兄弟市和海内外友好人士，表示衷心的感谢！

我们也清醒地看到，芜湖经济总量不够大、产业结构不够优、城市综合竞争力不够强的问题依然突出，“十二五”发展速度虽然领先全省，但并未达到预期；城市功能和品质需要进一步提升，城市管理不够精细，安全生产形势严峻；发展要素集聚不足，人才队伍规模不大，特别是高层次人才紧缺；资源约束趋紧，环境治理任务较重；公共服务供给不足，居民收入不高，人口老龄化加快，部分群众仍未脱贫；文明创建、法治建设有待加强；政府工作人员能力水平有待提高，少数工作人员作风亟待转变。对此，我们一定高度重视，采取措施切实加以解决。

二、“十三五”时期的奋斗目标和任务

“十三五”时期，是全面建成小康社会的决胜期和弯道超越的关键期。当前，世界经济在深度调整中曲折复苏，新一轮科技革命和产业变革蓄势待发。我市产业基础良好，近几年基础设施和交通条件明显改善，又面临国家“一带一路”、“中国制造2025”、“互联网+”、长江经济带、加强供给侧结构性改革等重大战略机遇，只要我们准确把握新常态下经济发展新特征，聚焦有限目标，奋力抢占行业制高点，同时用好资本市场，就一定能推动经济保持中高速增长、制造业率先迈向中高端水平，在提升城市核心竞争力上迈出更大步伐。

“十三五”时期发展的指导思想是：高举中国特色社会主义伟大旗帜，全面贯彻党的十八大和十八届三中、四中、五中全会精神，以马克思列宁主义、毛泽东思想、邓小平理论、“三个代表”重要思想、科学发展观为指导，深入贯彻习近平总书记系列重要讲话精神，紧紧围绕“四个全面”战略布局，坚持发展是第一要务，坚持创新、协调、绿色、开放、共享五大发展理念，以提高发展质量和效益为中心，加快调结构转方式促升级，打造具有国际竞争力的先进制造业基地、国家级旅游目的地、重要的综合交通枢纽、现代物流中心和长江经济带具有重要影响的现代化大城市，建设创新优美和谐幸福新芜湖，力争高水平全面建成小康社会。

主要思路是：认真落实五大发展理念和省调结构转方式促升级战略部署，坚持“向东看、往东比”，突出人才优先发展主战略，着力打造经济和城市两个升级版，努力走出一条人才结构高端化、产业层次高端化、城市品质高端化良性互动的内涵式发展路子，奋力实现弯道超越。

主要目标是：地区生产总值4300亿元，向4500亿元冲刺，经济增速居全省及长三角城市前列。常住人口城镇化率达65%。发展质量效益、城市功能品质、生态环境质量、人民生活水平和社会文明程度、制度体系建设水平显著提高。

突出把握好以下五个方面：

第一，实施创新发展，打造特色鲜明的国家创新型城市。坚持把创新摆在发展全局的核心位置，实施人才优先发展战略，广泛集聚创新资源。

提升人才特区建设水平。健全引进创新创业人才和团队政策体系、全程服务体系。到2020年，引进培育创新创业领军人才200人、创新创业团队300个。

加快创新型城市建设步伐。大力培育科技型企业，支持研发机构建设，发展高新技术产业。推进政产学研合作。实施科技企业孵化器提升工程。推进创新服务平台建设。加强知识产权保护。

聚焦培育战略性新兴产业。重点是机器人及智能装备、新型显示及光电信息、现代农业机械、新能源汽车、通用航空、节能环保装备、新材料、生命健康、轨道交通装备等产业。打造国内一流的工业机器人、新能源汽车、节能环保装备、新材料产业基地，建设全球重要的农机研发制造、通用航空、新型显示基地，成为全省新兴产业发展排头兵。

推动支柱产业转型升级。大力发展智能制造，推动奇瑞、海螺进军世界500强，支持美的、格力、新兴铸管等龙头企业做大做强。

加快发展现代服务业。争创国家快递示范园区，加快皖江航运服务业集聚区、保税物流园区建设，打造全国重要的现代物流中心。建成国家级电子商务示范城市和区域性电子商务中心。加快发展生活性服务业。推进金融创新，完善业态，拓展功能，打造区域性金融中心。上市公司超过30家，力争40家，“新三板”挂牌企业超过120家，力争150家，直接融资净额超过1200亿元。依托皖南国际文化旅游示范区平台，提升“欢乐芜湖”旅游目的地形象。建设国家级动漫产业基地和数字出版基地。到2020年，建成10个高水平主题公园。服务业比重力争达50%。

大力发展现代农业。推进农村综合体制改革，建设一批国家、省级现代农业示范区和农产品加工示范园区，大力发展农业服务业，打造现代农业强市。

全面深化改革。推进行政管理、财税、公共资源交易监管、国企国资等改革。大力扶持民营经济，发展混合所有制经济。

第二，实施协调发展，建设现代化滨江大城市。正确处理城乡、区域、物质文明与精神文明等重大关系，实现城乡一体、协调发展。

建设综合立体交通体系。建成商合杭铁路、皖赣铁路新建双线、庐铜铁路，完成芜铜、皖赣、淮南线铁路电气化改造，争取开工建设北沿江高铁、铜南宣铁路，打造区域性综合铁路枢纽中心。建成芜黄高速公路南陵段、北沿江高速无为段、岳武高速东延段，实施合芜、芜宣、芜马高速公路改扩建等项目。将芜湖港打造成安徽水上门户、长江中下游江海多式联运综合枢纽和上海国际航运中心的重要喂给港。民航机场建成通航。

着力完善城市功能。强化规划引领，推进“多规合一”。完成城市交通提升六年行动计划，打通断头路，健全微循环，拓展出入口，构建快速网。有序推进城市轨道交通规划建设，建成1号线和2号线一期。

积极推进跨江发展。支持江北产业集中区加快发展。加快芜湖长江二桥、商合杭大桥、城南过江隧道等通道建设。提升大龙湾新型城镇化示范区建设水平。支持无为县加快城市化进程。

加快智慧城市建设。建设公交都市。推进地下综合管廊和海绵城市建设。推进城市修补和有序更新，完成城市规划区范围内棚户区改造。

深入推进以人为核心的新型城镇化。统筹城乡发展，做强县域经济。争取无为、南陵撤县设市，芜湖县、繁昌撤县设区。以提升市民文明素质为重点，加强精神文明建设。推动文化事业繁荣。创建军民融合创新示范产业园区，推动军民融合深度发展。

第三，实施绿色发展，建设美丽芜湖。加强生态保护和环境治理，大力发展低碳循环经济，使芜湖的天更蓝、地更净、水更清、山更绿、空气更清新。

落实主体功能区规划，加强生态空间管控。推进资源和能源节约，严禁新建高耗能高污染项目、产能过剩项目。加强水、土地、矿产等资源节约集约利用。加大环境保护和污染治理力度。推进经济园区循环化改造，大力发展低碳循环经济。到2020年，新增绿地2000万平方米，森林覆盖率达20%，市区污水处理率达95%。争创国家环保模范城市和生态文明先行区、国家生态园林城市、国家森林城市、全国水生态文明城市。

第四，实施开放发展，打造沿江开放新高地。积极主动融入国家开放战略，全面提高开放型经济水平。

营造进一步扩大开放的环境。提升综合保税区等平台建设水平，复制推广自贸试验区经验，积极申建自贸试验区。按照国际先进水平提升城市服务功能。加强与欧美等发达国家及港澳台地区合作。鼓励企业参与“一带一路”建设，深化对内对外开放合作。

促进对外贸易优化升级。积极申报进口指定口岸。加快发展跨境电子商务，推进口岸直销中心和期货保税交割平台建设。加快形成出口基地集群。

第五，实施共享发展，建设幸福芜湖。加大公共事业投入，更加注重社会建设，促进各项公共服务均等化、标准化。

打造教育名城。合理配置教育资源，扩大优质教育资源供给，培育一批名教师、名校长、名学校。促进教育公平，全面普及15年基础教育，在全省率先实现教育现代化。

打造皖江医疗中心。持续深化医药卫生体制改革，建立覆盖城乡的基本医疗卫生制度和现代医院管理制度。全面实施一对夫妇可生育两个孩子政策。建设多层次养老服务体系。积极争创国家卫生城市、全国食品安全城市。

建立更加公平、更可持续的社会保障制度。持续增加城乡居民收入。争取2017年现有7.1万贫困人口全部脱贫。运用法治思维和法治方式推动发展。牢固树立安全发展观念，严格落实安全生产责任制。提高社会治理体系和治理能力现代化水平。

三、2016年的主要工作

2016年是实施“十三五”规划的开局之年。今年经济社会发展的主要预期目标是：地区生产总值比上年增长10%，居民收入同步增长，财政收入增长9%，城镇新增就业6.5万人，城镇登记失业率4.5%以内，节能减排完成省政府下达任务。

为实现上述目标，我们将大力去产能、去库存、去杠杆、降成本、补短板，着力抓好以下工作。

（一）保持经济平稳较快发展。

狠抓国家宏观调控和省、市相关政策落实。完善“一企一组一策”帮扶机制，优化市企业服务中心服务平台运行机制。促进企业上市、挂牌、增发、发债，利用政银担合作机制、税融通、过桥贷款、股权投资基金等方式支持实体经济发展。争取3家企业首发上市，新增“新三板”挂牌企业30家，直接融资220亿元。推广运用政府和社会资本合作模式。

转变招商思路，从单纯的资金引进转变为引资、引智、引技相结合，从传统的“政策让利”转变为项目投入提供更专业、更高效的公共服务上来，项目融资从单一的银行贷款转变为多层次资本市场运作，促进各类优质资源要素集聚。一个县区、一个开发区聚焦一个产业，站在行业的制高点上，推进重大项目的招引、落地。围绕交通、生态、健康、水利等重大工程领域，谋划、招引一批重大项目。持之以恒推进重大项目建设。

（二）大力推动产业转型升级。

推进战略性新兴产业基地建设。出台战略性新兴产业集聚发展基地建设专项引导资金管理办法。扩大机器人、新型显示2个国家战略性新兴产业区域集聚发展试点成果，加快建设3个省战略性新兴产业集聚发展基地。加快通用航空、新型显示及光电信息、新材料、文化科技等产业的发展，积极申报国家和省战略性新兴产业集聚发展基地。开工建设海创节能板材二期、信瑞新能源基地、安川电机、初级教练机、庞巴迪单轨车辆等项目，加快新能源汽车产业研究院、无为和三山智慧农业生产基地、信义电子超薄玻璃等项目建设进度，建成中电科钻石飞机、赛宝新能源汽车公共检测平台、奇瑞电动汽车增程器改扩建等项目。

加快传统产业改造提升。全面落实省调转促“4105”行动计划。运用先进技术，改造提升汽车及装备制造业、材料、电线电缆业，壮大四大支柱产业。支持奇瑞、海螺、美的、新兴铸管等龙头企业新上一批重大技术改造项目。将转型升级与淘汰落后产能、兼并重组相结合，完善产能过剩行业产能减量置换方案，启动50家企业兼并重组。

加快服务业发展。培育养老家政健康、信息、旅游休闲、绿色、住房、教育文化体育等消费增长点。发展工业设计、现代物流、金融服务等生产性服务业。积极申建民营银行。启动物联网医学产业园项目，加快老船厂?智慧港、新芜电商产业园、国家级广告产业园等载体建设。启动“十分钟快递便民服务圈”工程。支持三只松鼠、宇培吉品、长信智控、阡陌科技、外贸供应链、共生物流等龙头企业发展。加快方特复兴之路、新华联等项目建设。开工建设芜湖旅游集散中心。

加快农业现代化建设。开展好第三次全国农业普查和农村集体资产股份制改革试点。大力发展特色农业、设施农业、智慧农业，以农机现代化推动农技、农艺、农资、农信的新融合。推进农村金融综合改革。建成青弋江分洪道、澛港闸和荆方、双摆渡排涝泵站主体工程，开工建设青弋江治理、裕溪河治理工程。扎实推进“三线三边”整治、美丽乡村建设。

（三）努力提升城市品质。

坚持规划引领，推进新型城镇化。加快中心城区各组团服务配套设施提升、“双核”城市功能布局等课题研究。编制城乡建设系统“十三五”综合发展、城市近期建设规划。完成全市“多规合一”规划，探索空间开发建设管理模式。全面开展城市设计。完成城市景观风貌规划，制定城市风貌控制导则。建立三维城市信息平台，形成建筑三维审查的管理模式。提升县域规划建设管理水平。

完善综合交通体系，改善城市基础设施。开工建设新火车站西站房（西广场）、城市轨道交通2号线，建成庐铜铁路、芜铜铁路改建工程。开工建设宁芜高速芜湖东出入口、综合客运枢纽、米市口立交、马塘立交、淮九路扩宽改造项目，建成和谐大道、澛港闸大桥、205国道南陵渡大桥工程。优化城市交通，完成中江桥、中山桥老桥拆除，新桥开工建设；积极申报国家海绵城市试点市，推进省海绵城市试点项目长江大桥南岸桥公园建设；开工建设长江路地下综合管廊、沿河北路；建成3处地下公共停车场。启动野生动物园、滨江公园三期建设，新增绿地400万平方米，新建绿道80公里。完成造林7.3万亩。加快江北新型城镇化步伐。完善城东新区、新兴产业基地周边生活服务设施。推进高层住宅二次供水改造。加强取用水监管。实施329国道芜湖县段拓宽及备用水源工程。开工建设保兴垾二期、城市防洪墙改造，确保安全度汛。

强化住房保障。完成棚户区改造300万平方米，提高改造资金转化率。落实房地产去库存化政策措施，完善商品房预售资金监管，加快户籍制度改革，鼓励农民进城购房，促进房地产市场平稳健康发展。加快建筑产业现代化示范项目建设。

着力打造智慧城市，促进城市治理体系和治理能力现代化。突出城市空间和人文特色塑造，保护和传承历史、地域文化。发挥大数据效用，完善城市管理“一站通”行政权力运行平台。推进老旧小区改造、老旧市场搬迁改造。提升城市美化、亮化水平。探索物业服务新模式。

（四）不断深化改革开放。

加快推进城市服务管理信息化，发挥网格化管理优势，推行基层综合监管、分类执法模式。整合治安、城管、民政等基层队伍，运用“社管通”平台开展巡查，完善事中事后闭环监管体系。

加大国有企业改革力度，国有独资企业进人“逢进必考”。加快诚信体系建设。开展企业注册全程电子化试点，建设企业信用信息公示系统。深化预算管理制度改革，规范转移支付制度。强化政府性债务管理，完善政府财务报告制度，加强存量资金清理，全面实行国库支付电子化。鼓励与规范互联网金融产业发展，加强金融风险防控。完善招标采购评价办法，优化市县一体化公共资源交易平台功能。

深化医药卫生体制改革。推进计划生育服务管理创新。推进农村综合改革、国有林场改革和土地管理改革。开展国家深化工业用地市场化配置改革试点、矿产资源节约集约开发利用试点。

提高开放型经济水平。深化对外投资改革，落实以备案为主、核准为辅的对外投资管理办法。支持海螺、奇瑞境外项目建设。启动综合保税区扩区。推动开发区转型升级，努力把开发区建成产城一体的现代化新城区。深化中德合作，加快中欧国际物联网医学研究院等项目建设。积极参与长三角城市群分工与合作。加快芜马同城化，推进芜亳合作共建。强化皖江航运枢纽地位，推进与上海港的深度合作、与上游和周边港口的合作共赢。

（五）鼓励大众创业、万众创新。

推进人才特区、创业特区建设，支持支柱产业、战略性新兴产业、重点企业领军人才和团队招引。建设国际人才社区。加快众创空间建设，构建众创空间体系。举办创新创业大赛，建设市级创业产品展示中心和青年创业产品定向招标采购目录库。完善创业投融资机制。支持企业提升研发能力，新增高新技术企业40家。加快产业技术研究院等公共研发平台发展。加强市科技创新服务中心技术转移、大型仪器设备共享等公共服务平台建设，创新产学研人员双向流动合作的新机制。加快科技企业孵化器建设，推广“持股孵化”、“孵化+创投”等孵化模式。大力引进各类人才团队，优化人才服务环境，鼓励人才在芜湖创新创业。启动企业家培养计划。

（六）推进生态文明建设。

实施一批减排重点项目。组织重点污染行业实施技术改造。推进清洁生产，实现由末端治理向污染预防的转变。坚决落实大气、水污染防治行动计划。抓好饮用水源地综合整治。加快餐厨废弃物和污水处理设施建设进度，实行市区污水管网统一管理。

强化节能目标考核，实施一批节能重点项目。推进企业节能低碳行动。加强节能监测，控制能源消费增量。开展重点单位温室气体直报系统填报工作，探索开展碳排放权交易。力争开工建设经开区天然气分布式能源站，建成中电环保发电一期、绿洲环保垃圾焚烧发电厂改扩建、无为生物质发电项目。争创国家循环经济、资源综合利用、低碳城市等试点示范。

落实用地“双向约束”、用地规模预核定等政策，强化耕地保护。清理盘活存量用地、闲置土地。探索制定市级绿色矿山标准。

（七）发展各项社会事业。

投入99.6亿元，实施33项民生工程。开展精准扶贫。

完善就业服务体系。加快皖江人力资源公共实训基地建设，强化就业失业动态监测，推进校企合作和高校就业联盟工作。完善城乡就业困难人员援助制度，统筹做好高校毕业生等群体就业。稳妥推进城乡居民医保统筹，积极探索与职工医保“三保合一”。不断扩大社会保险覆盖范围，继续实施社会保险费减负政策。实施好机关事业单位养老保险制度改革。实行定点医疗机构和定点零售药店协议管理，完善医疗保险费用按病种分值结算办法，建立住院医疗费用互审制度。完善生育保险政策。推进工伤预防、补偿和康复“三位一体”体系建设。发展慈善事业。

提升教育和医疗服务水平。大力推进集团化办学，优化教育资源配置，力争新增教育集团5个、优质学校办分校（分园）3个、结对帮扶学校（幼儿园）30对。力争华师大外国语实验学校招生。完成技师学院新校区二期工程。推进教育信息化基础设施建设，建设覆盖各级各类学校的宽带网络和资源平台。加快职业教育人才培养模式改革，大力推广双元制职业教育，启动中德职业教育跨国培训合作项目，推进校企联合招生、现代学徒制度试点。支持在芜高校发展，开工建设国际工程师学院。合理配置医疗资源，完善基层医疗卫生机构运行机制。推动形成基层首诊、上下联动的分级诊疗机制。落实生育政策，改进计生服务。

积极发展文化事业和文化产业。坚持用中国梦和社会主义核心价值观凝聚共识、汇聚力量，弘扬优秀传统文化。以守法、诚信为主题，引导、规范市民公共行为，着力提高市民文明素质。发展文学艺术、新闻出版、广播电视等事业。加快实施芜湖百年建筑复活计划。开展近代西洋建筑、历史影像资料、新发现文物保护修复等研究。申报国家历史文化名城。实施全民健身苑、农民体育健身工程。开工建设奥林匹克公园二期，完成神山、赭山公园全民健身体育设施建设。举办第七届中国科普产品博览交易会。巩固城市民族工作成果，做好民族、宗教、外事、侨务等工作，发展档案、地方志、人防、气象、防灾减灾等各项事业。完善养老服务体系，鼓励社会投资养老服务业。

（八）着力维护公共安全和社会稳定。

继续把安全生产和公共安全管理作为头等大事，树立隐患即事故的理念，推进 “铸安”行动，强化安全隐患排查整治，严格落实责任制，强化公共安全和食品药品安全宣传教育，坚决防止各类事故发生。完善重大事件应急处置方案，做好处置演练。启动公安视频监控“天网工程”二期建设。强力打击传销活动。强化司法保障，依法防范和严厉惩治违法犯罪活动。

支持群团组织、社会组织依法参与社会治理。启动“七五”普法工作。支持残疾人事业发展。完善价格监测预警和信息发布机制。进一步完善信访制度，推行“阳光信访、责任信访、法治信访”。

支持驻芜部队建设，做好国防教育、优抚安置和双拥共建工作，增强全民国防意识。推进优势民营企业进入军工军品生产行列和“军转民”。

各位代表！

完成“十三五”时期的目标任务，对政府自身建设提出了更高的要求。面对新任务新要求，我们必须做到政治过硬、本领过硬、作风过硬、廉洁过硬，努力建设人民满意的政府。

建设法治政府。运用法治思维和法治方式提高行政能力。在政府决策、执行、监督全过程以及政府管理各个环节深入推进依法行政。认真执行市人大及其常委会的决议和决定，坚持重大决策出台前向人大报告。认真落实政府协商制度。自觉接受市人大、市政协监督，尊重司法监督，重视社会公众和舆论监督。认真办理人大代表议案、建议和政协委员提案，广泛听取各民主党派、工商联、无党派人士和工会、共青团、妇联等人民团体的意见，促进科学、民主决策。

建设服务政府。坚持简政放权、放管结合、优化服务，减少对微观经济活动的干预，建立事中事后监管体系。推进政务公开。提高政务效能，实行政府服务事项网上受理、审批和办结，明确办理时限，全程电子监察，发现问题严肃问责。巩固群众路线教育实践活动成果。建立健全政府工作人员考核机制，深入开展“双联系”、“亲切服务”、政风行风评议、民主评议科长等活动，下决心解决政务服务中态度不好、效率不高等问题，提升群众和企业办事满意率。

建设廉洁政府。严格执行中央八项规定和省、市相关规定精神，杜绝新建、购置楼堂馆所，严格控制机构和人员编制，从严控制和压缩“三公经费”。加强政府内部权力制约和制度建设，突出简政放权，推行分事行权、分岗设权、分级授权，把权力关进制度的笼子。加强行政监察，发挥审计监督作用，对公共资金、公共资源、国有资产严加监管。从解决群众反映强烈的问题入手，加大对发生在群众身边不正之风和腐败问题集中整治力度，保持惩治腐败的高压态势。完善责任追究制度，对发生重大腐败案件和严重违纪行为的，既追究当事人责任，又追究相关领导责任。

各位代表！

蓝图已经绘就，任务已经明确，关键在于苦干，根本在于落实。在新的历史起点上，我们要坚持“向东看、往东比”，以超前的思维、扎实的作风，高质量高效率地抓好每一个项目、推进每一项改革、落实每一项工作，奋力实现弯道超越，为建设创新优美和谐幸福新芜湖、高水平全面建成小康社会而努力奋斗！
